# London

**Published:** 1907-09-07

**Authors:** 


### NOTES ON MEDICAL SCHOOLS AND COLLEGES.

### LONDON.

*St. Bartholomew's Hospital Medical School.*—St. Bartholomew's is the oldest second largest hospital in London; the site has been recently enlarged by the purchase of 1½ acre of adjoining ground, and the first block of new buildings erected at the cost of £130,000 was recently opened by His Royal Highness the Prince of Wales, K.G. These buildings comprise casualty and out-patient departments, eight special departments, new quarters for the resident staff, a dining hall and common room for students, etc.

A new pathological institute is now in course of erection, and will be opened in the course of a year.

The erection of this block does not interfere in any way with the work of the hospital or the school.

*Central London Ophthalmic Hospital, Gray's Inn Road, W.C.*—This hospital has twenty-six beds, and possesses facilities for daily clinical teaching. Last year there were 355 in-patients and 12,910 out-patients (entailing 31,230 attendances). Classes of instruction on the following subjects will be held during the winter session, commencing in October. The course is open to both men and women students, and those wishing to attend are requested to send in their names to the Dean. The use of the ophthalmoscope, fee £2 2s. Errors of refraction, fee £1 1s., Mr. Ernest Clarke. Medical ophthalmology, fee £1 1s., Dr. C. O. Hawthorne. Operative surgery and surgical anatomy, fee £2 2s., Mr. Hancock. Pathology of the eye, fee £2 2s., Mr. Mayou. *X*-ray instruction, fee £1 1s., Mr. Mayou. Clinical lectures on external diseases of the eye. The post of clinical assistant at the hospital is open to both men and women, who must be qualified and registered practitioners. A composition fee of £5 5s. will entitle students to a perpetual ticket, and £3 3s. to three months' hospital practice. For syllabus and further particulars apply to the Dean.

*Charing Cross Hospital.*—This hospital contains 287 beds, and has attached to it a medical school. Classes for University, Conjoint, and Fellowship courses are held, and there are special classes for the practical work required for the D.P.H. course. Total fees, including students' club: For general students: (1) Composition fee 115 guineas; (2) sessional payments: Entrance fee 10 guineas and 15 guineas at the commencement of each winter and 10 guineas at the beginning of each summer session so long as the student remains in the school. The usual resident and students' appointments are open to students, and several valuable prizes and scholarships are awarded annually by the school.

*Guy's Hospital.*—This hospital contains 602 beds, and has a large medical school. There are special departments, and the number of students' appointments available is probably larger than in other metropolitan hospitals. Special classes are held for London University, Cambridge and Fellowship courses. Students have the advantage of a large and well-equipped residential club. For further particulars application should be made to the Dean of the Medical School, Dr. H. L. Eason.

*London (Royal Free Hospital) School of Medicine for Women.*—The Royal Free Hospital is in Gray's Inn Road, and the London School of Medicine for Women is in Hunter Street, Brunswick Square, W.C.

There are 165 beds in the hospital, all of which are available for clinical teaching.

There are numerous scholarships and prizes offered annually in connection with the school, among which may be mentioned the School Scholarship of £30 and the St. Dunstan's Medical Exhibition of £60 a year for three years, extendible to five years.

*The London Hospital Medical College.*—The London Hospital is the largest institution of its kind in England, and being situated in the East End, it ministers to a very large and poor district.

Seven entrance scholarships are offered annually, including the Price Scholarship in science, £120; the Epsom Scholarship, £126; and entrance scholarships in science, anatomy and physiology, and arts.

The composition fee is 120 guineas if paid in one sum, or 130 guineas if paid in three annual instalments. A reduction of 15 guineas is allowed in the case of sons of medical men.

Full information may be obtained from the Warden of the Medical School.

*The Middlesex Hospital Medical School.*—The hospital, which is in Mortimer Street, W., contains 340 beds, including special wards for children and the diseases of women. There is a cancer wing which has 40 beds, and special research laboratories, and the hospital therefore offers unequalled opportunities for the study of this disease, both clinically and pathologically.

A complete electrical and *x*-ray department has been provided in a temporary building which has been erected in the courtyard of the hospital.

*King's College Hospital.*—King's College Hospital, which contains 220 beds, is situated in Portugal Street, Lincoln's Inn, and is about five minutes' walk from King's College.

The new hospital, which is now being built in the neighbourhood of Denmark Hill, will be on a much larger scale than the present institution, and will possess every modern requirement for both patients and students. The teaching of the preliminary subjects, including chemistry, physics, biology, anatomy, and physiology, continue to be given at King's College in the Strand, and students entering now may by the time they are sufficiently advanced to study in the wards be able to do so in the new hospital.

The composition fee is—for advanced medical studies—70 guineas, for University course, 140 guineas; for whole Conjoint course, 135 guineas. Full particulars and prospectuses can be obtained by applying to Peyton Beale, Esq., F.R.C.S., Dean of the Hospital, or to Professor Peter Thompson, King's College, Strand.

*St. George's Hospital.*—The winter session commences on October 1, but students can enter at any time. Special courses are given, in which the requirements of university and other examinations receive careful attention. The hospital contains 350 beds, and the usual resident and students' appointments are open to students. Further particulars may be obtained on application to the Dean of the Medical School, Dr. E. I. Spriggs.

*St. Mary's Hospital Medical School.*—St. Mary's Hospital at present contains 281 beds, which number will be raised to 341 on the opening of the Clarence Wing. This extension will also provide new operating theatres, a medical clinical theatre, an obstetric department for cases of difficult labour, and a complete electrical department. A new *x*-ray department has recently been opened in the out-patients' department.

The composition fee is £140 if paid in one sum on entering the Medical School, and £145 if paid in four instalments. On payment of the full composition fee, and provided that he obtains a registrable medical qualification within seven years from the date of registration, the student becomes a perpetual pupil, and is entitled to unlimited attendance on the practice of the hospital. The composition fee for students who have passed their examination in anatomy and physiology is 60 guineas if paid at entrance, 65 guineas if paid in two annual instalments.

*University College Hospital Medical School* comprises the departments of medicine and clinical medicine, surgery and clinical surgery, midwifery and gynæcology, pathology and morbid anatomy and clinical pathology, bacteriology, mental physiology, and mental diseases, dental surgery, practical pharmacy, and other departments for the study of special diseases, such as those of the eye, skin, ear and throat, and for instruction in the use of anæsthetics, and in electro-therapeutics and the application of the *x*-rays.

Scholarships and exhibitions to the value of about £450 are offered for competition every year.

Composition fees for the courses required by the University of London: For the Final M.B., B.S. course, 80 guineas, or in two instalments of 50 and 32 guineas; for the medical education required by the Examining Board in England and the Society of Apothecaries, for the course required for the third examination, 80 guineas, or in two instalments of 50 and 32 guineas.

*University College (Faculty of Medical Sciences).*—The first term begins on October 1 next. Full courses are provided for students desirous of obtaining the medical degrees of the London University, as well as for those seeking the qualifications of other universities and licensing bodies. Facilities for research work are provided. A student can enter the college directly, and at the end of his preliminary courses select the medical school and hospital at which he intends to complete his course, or he can select his hospital and school at the outset, and enter the college for his preliminary course.

*Westminster Hospital Medical School.*—Westminster Hospital was the first institution of its kind in London which was founded by the voluntary contributions of the public. There is accommodation for upwards of 200 in-patients. There is a Students' Club Union, the subscription for membership of which is included in the general fees. The composition fee is 120 guineas for the course of the Conjoint Board, and 130 guineas for that of the University of London.

